# Crystal structure of (3*E*)-3-[(4-nitro­phen­oxy)­meth­yl]-4-phenyl­but-3-en-2-one

**DOI:** 10.1107/S1600536814018327

**Published:** 2014-08-16

**Authors:** Julio Zukerman-Schpector, Stella H. Maganhi, Paulo J. S. Moran, Bruno R. S. de Paula, Paulo R. Nucci, Edward R. T. Tiekink

**Affiliations:** a Departmento de Química, Universidade Federal de São Carlos, 13565-905 São Carlos, SP, Brazil; b Departmento de Física, Universidade Federal de São Carlos, 13565-905 São Carlos, SP, Brazil; c Instituto de Química, Universidade Estadual de Campinas, CP 6154, 13083-970 Campinas, SP, Brazil; d Programa de Pós Graduacão em Biotecnologia, Universidade Federal de São Carlos, 13565-905 São Carlos, SP, Brazil; e Department of Chemistry, University of Malaya, 50603 Kuala Lumpur, Malaysia

**Keywords:** crystal structure, hydrogen bonding, π–π inter­actions

## Abstract

In the title compound, C_17_H_15_NO_4_, the conformation about the C=C double bond [1.348 (2) Å] is *E* with the ketone group almost co-planar [C—C—C—C torsion angle = 7.2 (2)°] but the phenyl group twisted away [C—C—C—C = 160.93 (17)°]. The terminal aromatic rings are almost perpendicular to each other [dihedral angle = 81.61 (9)°] giving the mol­ecule an overall U-shape. The crystal packing feature benzene-C—H⋯O(ketone) contacts that lead to supra­molecular helical chains along the *b* axis. These are connected by π–π inter­actions between benzene and phenyl rings [inter-centroid distance = 3.6648 (14) Å], resulting in the formation of a supra­molecular layer in the *bc* plane.

## Related literature   

For background to biotransformations mediated by *Saccharomyces cerevisiae*, see: Rodrigues *et al.* (2004 ▶); de Paula *et al.* (2013 ▶).
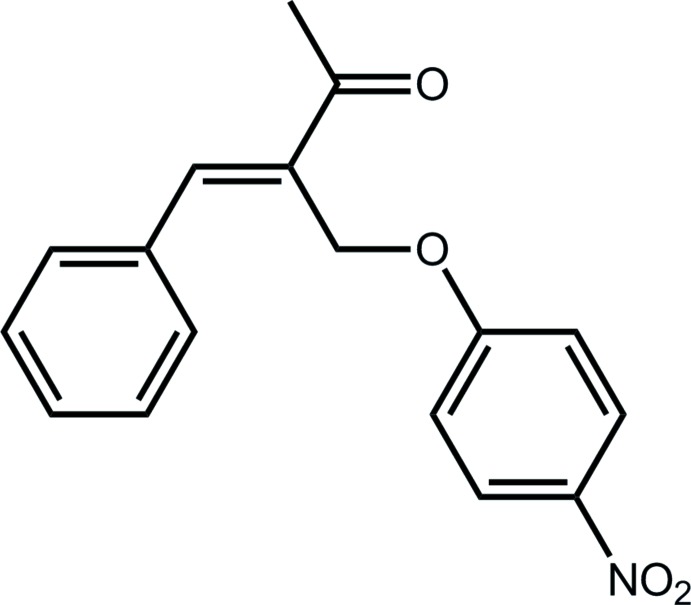


## Experimental   

### Crystal data  

C_17_H_15_NO_4_
*M* *_r_* = 297.30
Monoclinic, 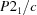

*a* = 12.769 (3) Å
*b* = 9.4607 (2) Å
*c* = 13.0022 (4) Å
β = 108.145 (1)°
*V* = 1492.6 (4) Å^3^
*Z* = 4
Mo *K*α radiation
μ = 0.10 mm^−1^
*T* = 290 K
0.52 × 0.23 × 0.12 mm

### Data collection  

Bruker Kappa APEXII CCD diffractometer
Absorption correction: multi-scan (*SADABS*; Sheldrick, 1996 ▶) *T*_min_ = 0.686, *T*_max_ = 0.745
9377 measured reflections
2667 independent reflections
2118 reflections with *I* > 2σ(*I*)
*R*_int_ = 0.021

### Refinement  

*R*[*F* ^2^ > 2σ(*F* ^2^)] = 0.038
*wR*(*F* ^2^) = 0.113
*S* = 1.06
2667 reflections
201 parameters
H-atom parameters constrained
Δρ_max_ = 0.16 e Å^−3^
Δρ_min_ = −0.12 e Å^−3^

### 

Data collection: *APEX2* (Bruker, 2009 ▶); cell refinement: *SAINT* (Bruker, 2009 ▶); data reduction: *SAINT*; program(s) used to solve structure: *SIR97* (Altomare *et al.*, 1999 ▶); program(s) used to refine structure: *SHELXL97* (Sheldrick, 2008 ▶); molecular graphics: *ORTEP-3 for Windows* (Farrugia, 2012 ▶), *DIAMOND* (Brandenburg, 2006 ▶); software used to prepare material for publication: *MarvinSketch* (Chemaxon, 2010 ▶) and *publCIF* (Westrip, 2010 ▶).

## Supplementary Material

Crystal structure: contains datablock(s) I. DOI: 10.1107/S1600536814018327/hg5405sup1.cif

Structure factors: contains datablock(s) I. DOI: 10.1107/S1600536814018327/hg5405Isup2.hkl

Supporting information file. DOI: 10.1107/S1600536814018327/hg5405Isup3.cml

**Fig. 1.**
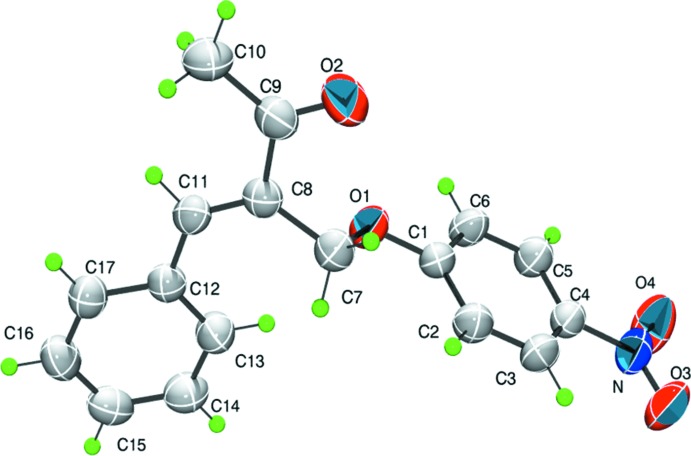
The molecular structure of the title showing the atom-labelling scheme and displacement ellipsoids at the 50% probability level.

**Fig. 2.**
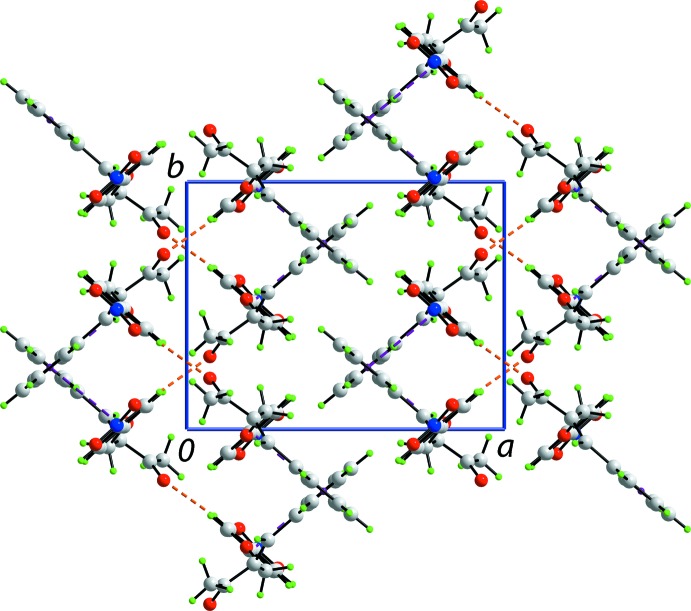
A view of unit-cell contents in projection down the c axis. The C—H···O and π—π contacts are shown as orange and purple dashed lines, respectively.

. DOI: 10.1107/S1600536814018327/hg5405fig1.tif
The mol­ecular structure of the title showing the atom-labelling scheme and displacement ellipsoids at the 50% probability level.

c . DOI: 10.1107/S1600536814018327/hg5405fig2.tif
A view of unit-cell contents in projection down the *c* axis. The C—H⋯O and π—π contacts are shown as orange and purple dashed lines, respectively.

CCDC reference: 1018885

Additional supporting information:  crystallographic information; 3D view; checkCIF report

## Figures and Tables

**Table 1:** Hydrogen-bond geometry (Å, °)

*D*—H⋯*A*	*D*—H	H⋯*A*	*D*⋯*A*	*D*—H⋯*A*
C5—H5⋯O2^i^	0.93	2.45	3.140 (2)	131

Symmetry code: (i) 
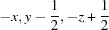
.

## supplementary crystallographic information

### S1. Structural commentary

As part of a continuing inter­est in biotransformations mediated by *Saccharomyces cerevisiae*, such as the bio-reduction of α-haloketones and enones (Rodrigues *et al.*, 2004), the title compound, (3*E*)-3-[(4-nitro­phen­oxy)­methyl]-4-phenyl­but-3-en-2- one, was synthesised to be used as a substrate in order to compare its behaviour with that of 3-halo­methyl-4-phenyl-3-buten-2-ones analogues (de Paula *et al.*, 2013). Herein, the crystal structure determination and spectroscopic details are described.

The conformation about the ethene bond in the title compound, Fig. 1, is *E*. The ketone group is almost co-planar with the double bond as seen in the C11—C8—C9—C10 torsion angle of 7.2 (2)° but the phenyl group is twisted away [C8—C11—C12—C17 160.93 (17)°]. The nitro group is co-planar with the benzene ring to which it is attached [O3—N—C4—C5 = 177.32 (17)°]. The terminal aromatic rings are almost perpendicular [dihedral angle = 81.61 (9)°] to each other so that overall the molecule has the shape of the letter U.

In the crystal packing, C5—H···O2 contacts, Table 1, lead to supra­molecular helical chains along the *b* axis and these are connected by π—π inter­actions between benzene and phenyl rings [inter-centroid distance = 3.6648 (14) Å; inter-planar angle = 2.70 (9)° for symmetry operation: *x*, 3/2-*y*, -1/2+*z*] to form a supra­molecular layer in the *bc* plane, Fig. 2.

### S2. Synthesis and crystallization

Potassium carbonate (1.66 g, 12 mmol) was added to a solution of 4-nitro­phenol (1.53 g, 11 mmol) and 3-bromo­methyl-4-phenyl-3-buten-2-one (2.39 g, 10 mmol) in acetone (10 mL). The reaction mixture was stirred for 6 h. Then, water (100 mL) was added and the product extracted with di­chloro­methane (3 x 50 mL). The organic layer was washed with brine, and dried over sodium sulfate. The solvent was removed under reduced pressure and the product purified by column chromatography (hexane/ethyl acetate, 8:2) to afford 3-[(4-nitro­phen­oxy)­methyl]-4-phenyl-3-buten-2-one as a colourless solid. The product was recrystallized by slow evaporation of a 1:4 mixture of hexane and di­chloro­methane. The crystallised isomer, was shown by crystallography to be the *E* isomer; M.pt: 398.6–399.6 K. ^1^H NMR (CDCl_3_, 400 MHz): δ 2.54 (3*H*, s), 4.93 (2*H*, s), 6.99-8.21 (9*H*, m), 7.92 (1*H*, s). ^13^C NMR (CDCl_3_, 100 MHz) δ 26.0, 62.3, 114.9, 125.9, 128.9, 129.6, 130.1, 134.1, 135.0, 141.8, 146.3, 163.5, 198.2. ESI±HRMS ((M+H)+) calcd.: 298.1079; found: 298.1049.

### S3. Refinement

Carbon-bound H-atoms were placed in calculated positions (C—H 0.93 to 0.97 Å) and were included in the refinement in the riding model approximation, with *U_iso_*(H) = 1.2–1.5*U_eq_*(C).

### Crystal data

**Table:**

C_17_H_15_NO_4_	*F*(000) = 624
*M**_r_* = 297.30	*D*_x_ = 1.323 Mg m^−^^3^
Monoclinic, *P*2_1_/*c*	Mo *K*α radiation, λ = 0.71073 Å
Hall symbol: -P 2ybc	Cell parameters from 3883 reflections
*a* = 12.769 (3) Å	θ = 2.7–25.1°
*b* = 9.4607 (2) Å	µ = 0.10 mm^−^^1^
*c* = 13.0022 (4) Å	*T* = 290 K
β = 108.145 (1)°	Irregular, colourless
*V* = 1492.6 (4) Å^3^	0.52 × 0.23 × 0.12 mm
*Z* = 4	

### Data collection

**Table:**

Bruker Kappa APEXII CCD diffractometer	2667 independent reflections
Radiation source: fine-focus sealed tube	2118 reflections with *I* > 2σ(*I*)
Graphite monochromator	*R*_int_ = 0.021
ω and φ scans	θ_max_ = 25.2°, θ_min_ = 1.7°
Absorption correction: multi-scan (*SADABS*; Sheldrick, 1996)	*h* = −9→15
*T*_min_ = 0.686, *T*_max_ = 0.745	*k* = −11→8
9377 measured reflections	*l* = −15→13

### Refinement

**Table:**

Refinement on *F*^2^	Secondary atom site location: difference Fourier map
Least-squares matrix: full	Hydrogen site location: inferred from neighbouring sites
*R*[*F*^2^ > 2σ(*F*^2^)] = 0.038	H-atom parameters constrained
*wR*(*F*^2^) = 0.113	*w* = 1/[σ^2^(*F*_o_^2^) + (0.0515*P*)^2^ + 0.3426*P*] where *P* = (*F*_o_^2^ + 2*F*_c_^2^)/3
*S* = 1.06	(Δ/σ)_max_ < 0.001
2667 reflections	Δρ_max_ = 0.16 e Å^−^^3^
201 parameters	Δρ_min_ = −0.12 e Å^−^^3^
0 restraints	Extinction correction: *SHELXL*, Fc^*^=kFc[1+0.001xFc^2^λ^3^/sin(2θ)]^-1/4^
Primary atom site location: structure-invariant direct methods	Extinction coefficient: 0.0115 (17)

### Special details

**Table:**

Geometry. All s.u.'s (except the s.u. in the dihedral angle between two l.s. planes) are estimated using the full covariance matrix. The cell s.u.'s are taken into account individually in the estimation of s.u.'s in distances, angles and torsion angles; correlations between s.u.'s in cell parameters are only used when they are defined by crystal symmetry. An approximate (isotropic) treatment of cell s.u.'s is used for estimating s.u.'s involving l.s. planes.
Refinement. Refinement of *F*^2^ against ALL reflections. The weighted *R*-factor *wR* and goodness of fit *S* are based on *F*^2^, conventional *R*-factors *R* are based on *F*, with *F* set to zero for negative *F*^2^. The threshold expression of *F*^2^ > 2σ(*F*^2^) is used only for calculating *R*-factors(gt) *etc*. and is not relevant to the choice of reflections for refinement. *R*-factors based on *F*^2^ are statistically about twice as large as those based on *F*, and *R*- factors based on ALL data will be even larger.

### Fractional atomic coordinates and isotropic or equivalent isotropic displacement parameters (Å^2^)

**Table:**

	*x*	*y*	*z*	*U*_iso_*/*U*_eq_	
O1	0.18817 (9)	0.97520 (13)	0.36449 (8)	0.0543 (3)	
O2	0.07283 (12)	1.20609 (17)	0.47397 (12)	0.0841 (5)	
O3	0.28681 (13)	1.06055 (17)	−0.07967 (11)	0.0831 (5)	
O4	0.16374 (15)	0.90131 (18)	−0.12490 (11)	0.0920 (5)	
N	0.22052 (14)	0.98328 (17)	−0.05788 (12)	0.0623 (4)	
C1	0.19856 (12)	0.98711 (17)	0.26230 (12)	0.0448 (4)	
C2	0.27428 (14)	1.07139 (18)	0.23691 (13)	0.0522 (4)	
H2	0.3210	1.1291	0.2893	0.063*	
C3	0.28067 (14)	1.06981 (18)	0.13101 (14)	0.0538 (4)	
H3	0.3328	1.1257	0.1139	0.065*	
C4	0.21123 (13)	0.98717 (17)	0.05269 (12)	0.0483 (4)	
C5	0.13386 (14)	0.90499 (19)	0.07617 (13)	0.0541 (4)	
H5	0.0858	0.8499	0.0227	0.065*	
C6	0.12779 (14)	0.90476 (19)	0.18155 (13)	0.0528 (4)	
H6	0.0755	0.8485	0.1981	0.063*	
C7	0.24741 (14)	1.07108 (18)	0.44897 (12)	0.0503 (4)	
H7A	0.2314	1.1681	0.4251	0.060*	
H7B	0.3261	1.0557	0.4664	0.060*	
C8	0.21127 (13)	1.04372 (17)	0.54754 (12)	0.0473 (4)	
C9	0.11652 (14)	1.13064 (19)	0.55156 (14)	0.0559 (4)	
C10	0.07616 (17)	1.1245 (2)	0.64904 (16)	0.0726 (6)	
H10A	0.0178	1.1918	0.6405	0.109*	
H10B	0.1358	1.1466	0.7131	0.109*	
H10C	0.0492	1.0313	0.6555	0.109*	
C11	0.25497 (13)	0.94713 (18)	0.62529 (12)	0.0475 (4)	
H11	0.2227	0.9440	0.6803	0.057*	
C12	0.34410 (13)	0.84741 (17)	0.63703 (12)	0.0459 (4)	
C13	0.38193 (14)	0.80114 (19)	0.55154 (14)	0.0550 (4)	
H13	0.3484	0.8336	0.4815	0.066*	
C14	0.46774 (15)	0.7087 (2)	0.57213 (15)	0.0612 (5)	
H14	0.4936	0.6793	0.5162	0.073*	
C15	0.51684 (15)	0.65826 (19)	0.67625 (16)	0.0611 (5)	
H15	0.5762	0.5964	0.6894	0.073*	
C16	0.47913 (15)	0.69833 (19)	0.76078 (15)	0.0608 (5)	
H16	0.5117	0.6624	0.8300	0.073*	
C17	0.39348 (15)	0.79129 (18)	0.74136 (13)	0.0531 (4)	
H17	0.3673	0.8180	0.7977	0.064*	

### Atomic displacement parameters (Å^2^)

**Table:**

	*U*^11^	*U*^22^	*U*^33^	*U*^12^	*U*^13^	*U*^23^
O1	0.0564 (7)	0.0642 (7)	0.0451 (6)	−0.0149 (6)	0.0200 (5)	−0.0061 (5)
O2	0.0779 (10)	0.0976 (11)	0.0751 (9)	0.0393 (9)	0.0214 (7)	0.0182 (8)
O3	0.0911 (11)	0.0998 (11)	0.0756 (9)	−0.0136 (9)	0.0507 (8)	0.0119 (8)
O4	0.1264 (13)	0.1010 (12)	0.0552 (8)	−0.0303 (11)	0.0376 (8)	−0.0145 (8)
N	0.0741 (11)	0.0658 (10)	0.0540 (9)	0.0029 (9)	0.0300 (8)	0.0076 (8)
C1	0.0415 (8)	0.0499 (9)	0.0447 (8)	0.0009 (7)	0.0157 (7)	0.0023 (7)
C2	0.0462 (9)	0.0575 (10)	0.0518 (9)	−0.0100 (8)	0.0135 (7)	−0.0011 (8)
C3	0.0481 (9)	0.0578 (10)	0.0591 (10)	−0.0071 (8)	0.0221 (8)	0.0081 (8)
C4	0.0495 (9)	0.0511 (9)	0.0481 (8)	0.0033 (8)	0.0208 (7)	0.0057 (7)
C5	0.0525 (10)	0.0609 (10)	0.0489 (9)	−0.0106 (8)	0.0160 (7)	−0.0052 (8)
C6	0.0478 (9)	0.0622 (10)	0.0518 (9)	−0.0138 (8)	0.0203 (7)	−0.0042 (8)
C7	0.0500 (9)	0.0507 (9)	0.0492 (9)	−0.0047 (8)	0.0139 (7)	−0.0037 (7)
C8	0.0444 (9)	0.0505 (9)	0.0458 (8)	−0.0007 (7)	0.0124 (7)	−0.0064 (7)
C9	0.0490 (10)	0.0609 (11)	0.0543 (10)	0.0056 (9)	0.0111 (8)	−0.0054 (8)
C10	0.0601 (12)	0.0882 (15)	0.0759 (12)	0.0178 (11)	0.0303 (10)	−0.0007 (11)
C11	0.0457 (9)	0.0556 (9)	0.0421 (8)	−0.0020 (8)	0.0149 (7)	−0.0065 (7)
C12	0.0434 (9)	0.0458 (8)	0.0475 (8)	−0.0036 (7)	0.0127 (7)	−0.0038 (7)
C13	0.0553 (10)	0.0597 (10)	0.0502 (9)	0.0060 (9)	0.0166 (8)	−0.0012 (8)
C14	0.0585 (11)	0.0613 (11)	0.0695 (11)	0.0063 (9)	0.0282 (9)	−0.0033 (9)
C15	0.0475 (10)	0.0535 (10)	0.0817 (13)	0.0032 (8)	0.0193 (9)	0.0038 (9)
C16	0.0577 (11)	0.0582 (11)	0.0610 (10)	0.0024 (9)	0.0104 (8)	0.0111 (8)
C17	0.0568 (10)	0.0517 (10)	0.0506 (9)	−0.0025 (8)	0.0164 (7)	0.0007 (7)

### Geometric parameters (Å, º)

**Table:**

O1—C1	1.3804 (17)	C8—C11	1.348 (2)
O1—C7	1.4436 (19)	C8—C9	1.477 (2)
O2—C9	1.220 (2)	C9—C10	1.511 (3)
O3—N	1.217 (2)	C10—H10A	0.9600
O4—N	1.222 (2)	C10—H10B	0.9600
N—C4	1.479 (2)	C10—H10C	0.9600
C1—C2	1.371 (2)	C11—C12	1.449 (2)
C1—C6	1.391 (2)	C11—H11	0.9300
C2—C3	1.405 (2)	C12—C17	1.409 (2)
C2—H2	0.9300	C12—C13	1.412 (2)
C3—C4	1.368 (2)	C13—C14	1.362 (2)
C3—H3	0.9300	C13—H13	0.9300
C4—C5	1.364 (2)	C14—C15	1.388 (3)
C5—C6	1.396 (2)	C14—H14	0.9300
C5—H5	0.9300	C15—C16	1.383 (3)
C6—H6	0.9300	C15—H15	0.9300
C7—C8	1.514 (2)	C16—C17	1.364 (2)
C7—H7A	0.9700	C16—H16	0.9300
C7—H7B	0.9700	C17—H17	0.9300
			
C1—O1—C7	119.76 (12)	O2—C9—C8	117.72 (16)
O3—N—O4	121.10 (16)	O2—C9—C10	121.91 (16)
O3—N—C4	118.91 (16)	C8—C9—C10	120.37 (15)
O4—N—C4	119.97 (15)	C9—C10—H10A	109.5
C2—C1—O1	124.46 (14)	C9—C10—H10B	109.5
C2—C1—C6	119.02 (14)	H10A—C10—H10B	109.5
O1—C1—C6	116.50 (13)	C9—C10—H10C	109.5
C1—C2—C3	119.38 (15)	H10A—C10—H10C	109.5
C1—C2—H2	120.3	H10B—C10—H10C	109.5
C3—C2—H2	120.3	C8—C11—C12	130.43 (15)
C4—C3—C2	120.91 (15)	C8—C11—H11	114.8
C4—C3—H3	119.5	C12—C11—H11	114.8
C2—C3—H3	119.5	C17—C12—C13	118.71 (15)
C5—C4—C3	120.38 (15)	C17—C12—C11	116.61 (14)
C5—C4—N	119.08 (15)	C13—C12—C11	124.66 (14)
C3—C4—N	120.53 (15)	C14—C13—C12	119.73 (16)
C4—C5—C6	119.06 (15)	C14—C13—H13	120.1
C4—C5—H5	120.5	C12—C13—H13	120.1
C6—C5—H5	120.5	C13—C14—C15	120.28 (17)
C1—C6—C5	121.22 (15)	C13—C14—H14	119.9
C1—C6—H6	119.4	C15—C14—H14	119.9
C5—C6—H6	119.4	C16—C15—C14	121.17 (17)
O1—C7—C8	108.12 (13)	C16—C15—H15	119.4
O1—C7—H7A	110.1	C14—C15—H15	119.4
C8—C7—H7A	110.1	C17—C16—C15	119.14 (17)
O1—C7—H7B	110.1	C17—C16—H16	120.4
C8—C7—H7B	110.1	C15—C16—H16	120.4
H7A—C7—H7B	108.4	C16—C17—C12	120.90 (16)
C11—C8—C9	120.21 (15)	C16—C17—H17	119.5
C11—C8—C7	125.92 (15)	C12—C17—H17	119.5
C9—C8—C7	113.82 (14)		
			
C7—O1—C1—C2	10.0 (2)	O1—C7—C8—C9	−90.67 (16)
C7—O1—C1—C6	−171.34 (14)	C11—C8—C9—O2	−172.68 (17)
O1—C1—C2—C3	176.97 (15)	C7—C8—C9—O2	5.0 (2)
C6—C1—C2—C3	−1.7 (2)	C11—C8—C9—C10	7.2 (2)
C1—C2—C3—C4	1.1 (3)	C7—C8—C9—C10	−175.10 (16)
C2—C3—C4—C5	0.3 (3)	C9—C8—C11—C12	177.64 (16)
C2—C3—C4—N	−178.36 (15)	C7—C8—C11—C12	0.2 (3)
O3—N—C4—C5	177.32 (17)	C8—C11—C12—C17	160.93 (17)
O4—N—C4—C5	−4.2 (3)	C8—C11—C12—C13	−20.7 (3)
O3—N—C4—C3	−4.0 (3)	C17—C12—C13—C14	−2.8 (3)
O4—N—C4—C3	174.52 (17)	C11—C12—C13—C14	178.89 (16)
C3—C4—C5—C6	−1.0 (3)	C12—C13—C14—C15	1.0 (3)
N—C4—C5—C6	177.68 (15)	C13—C14—C15—C16	1.0 (3)
C2—C1—C6—C5	1.0 (3)	C14—C15—C16—C17	−1.3 (3)
O1—C1—C6—C5	−177.76 (15)	C15—C16—C17—C12	−0.6 (3)
C4—C5—C6—C1	0.4 (3)	C13—C12—C17—C16	2.6 (2)
C1—O1—C7—C8	174.07 (13)	C11—C12—C17—C16	−178.98 (16)
O1—C7—C8—C11	86.91 (19)		

### Hydrogen-bond geometry (Å, º)

**Table:**

*D*—H···*A*	*D*—H	H···*A*	*D*···*A*	*D*—H···*A*
C5—H5···O2^i^	0.93	2.45	3.140 (2)	131

Symmetry code: (i) −*x*, *y*−1/2, −*z*+1/2.
