# Supplementary figures and images for: Crystal structure of (3E)-3-[(4-nitro­phen­oxy)­meth­yl]-4-phenyl­but-3-en-2-one
Source: Acta Crystallogr Sect E Struct Rep Online. 2014 Aug 16;70(Pt 9):o1020–1. doi: 10.1107/S1600536814018327 (PMC4186193; doi:10.1107/S1600536814018327)

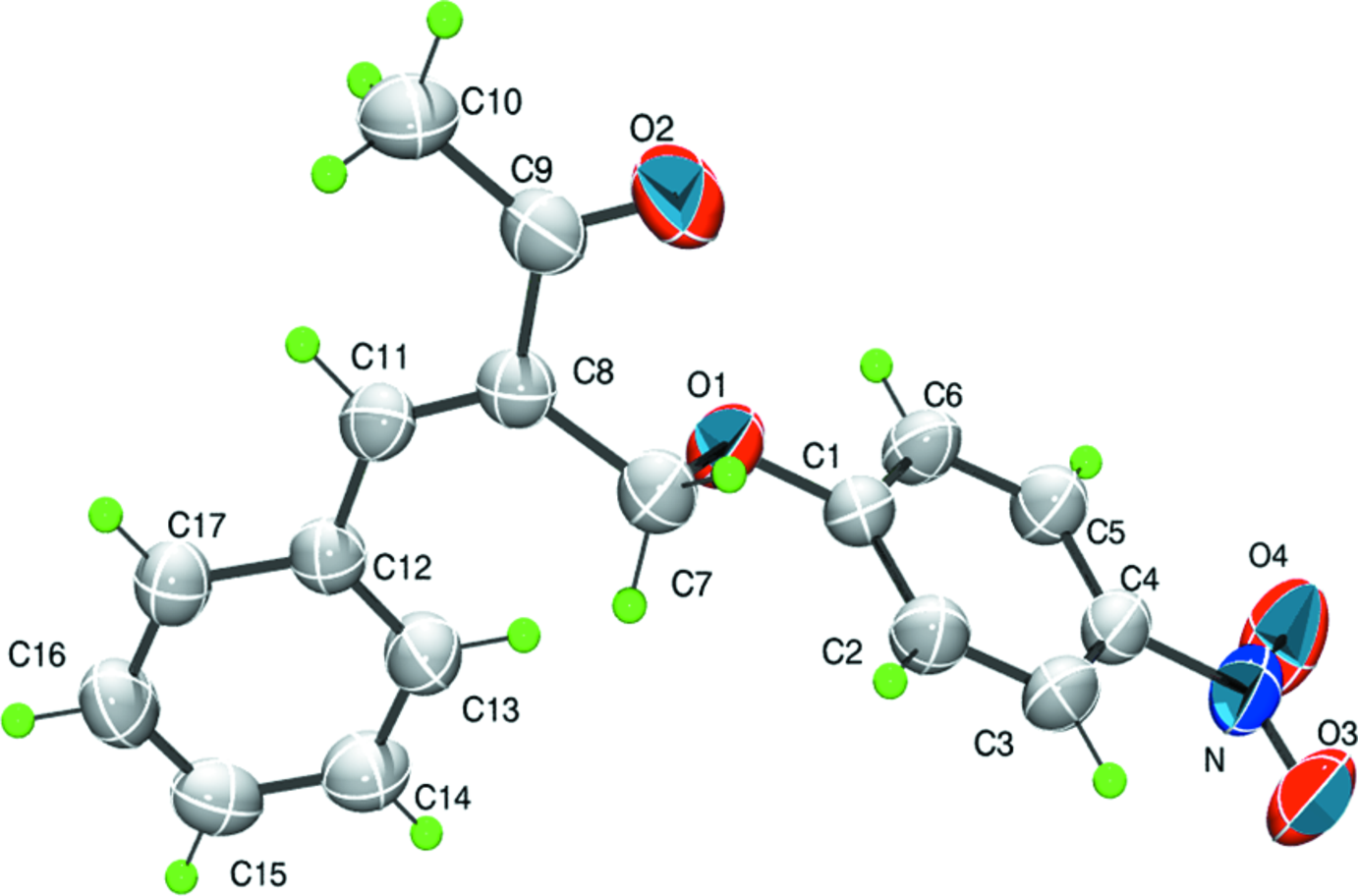

Supplement: Supplementary file 4 [file e-70-o1020-fig1.tif]

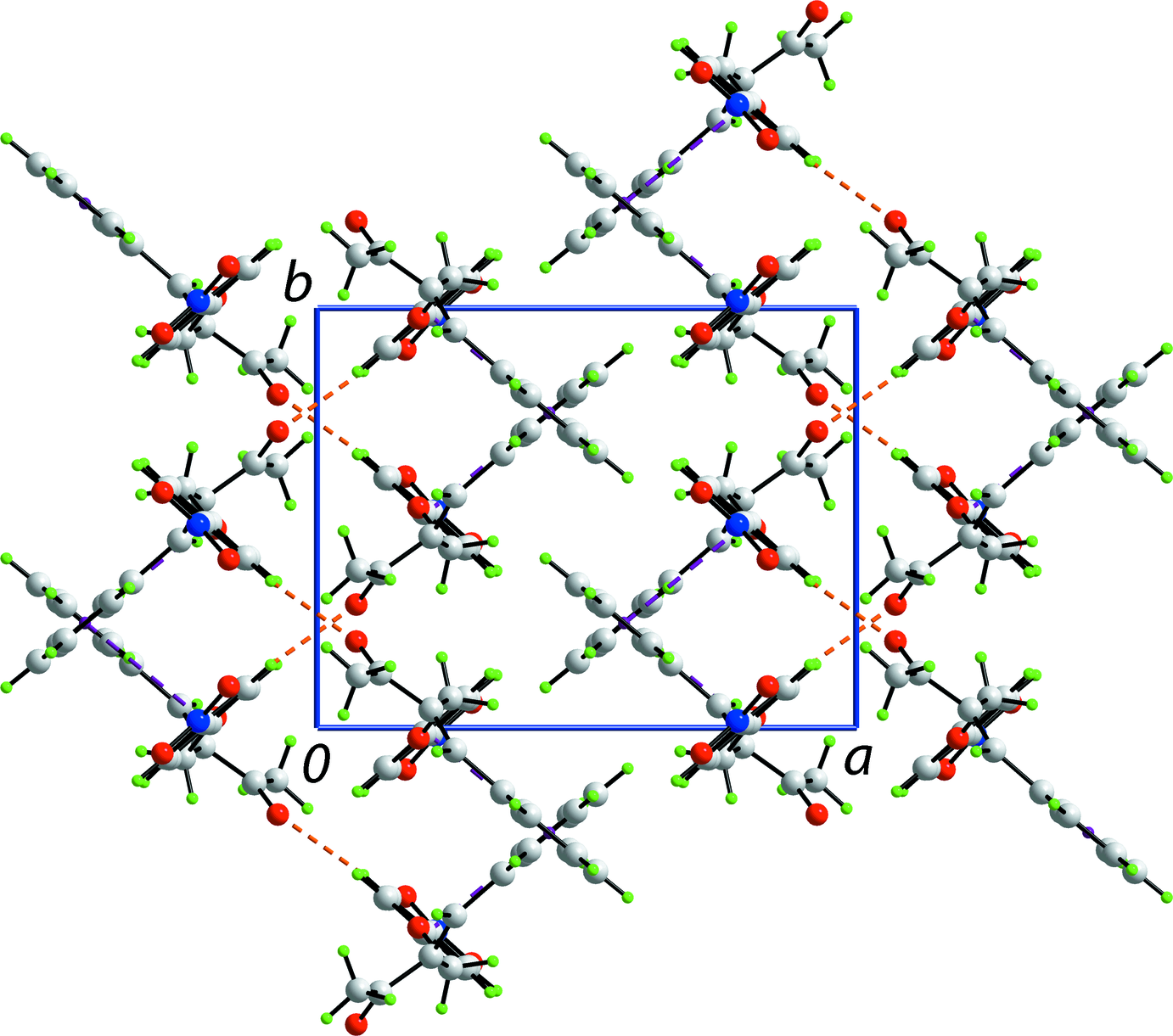

Supplement: Supplementary file 5 [file e-70-o1020-fig2.tif]
